# Supplementary material for: The Strength of Selection against Neanderthal Introgression
Source: PLoS Genet. 2016 Nov 8;12(11):e1006340. doi: 10.1371/journal.pgen.1006340 (PMC5100956; doi:10.1371/journal.pgen.1006340)
Supplement: S1 Text — Here, we describe several models of a single pulse of admixture between Neanderthal and modern humans, and derive approximations for the present-day frequency of a neutral introgressed Neanderthal allele linked to one or multiple sites under purifying selection in humans. We then demonstrate the accuracy of these approximations by comparing them to numerically iterated recursion equations and individual-based simulations. Lastly, we consider models of single and multiple waves of continuous introgression and show that one cannot distinguish between these models and a single-pulse admixture model using the present-day frequency of introgressed alleles as the only source of information. (PDF) [file pgen.1006340.s001.pdf]

S1 Text

Modeling Selection Against Introgression

Ivan Juric, Simon Aeschbacher, Graham Coop

Here, we describe several models of a single pulse of admixture between Neanderthals and modern humans, and derive approximations for the present-day frequency of a neutral introgressed Neanderthal allele linked to one or multiple sites under purifying selection in humans. We then demonstrate the accuracy of these approximations by comparing them to numerically iterated recursion equations and individual-based simulations. Lastly, we consider models of single and multiple waves of continuous introgression and show that one cannot distinguish between these models and a single-pulse admixture model using the present-day frequency of introgressed alleles as the only source of information.

Single-pulse introgression models

In the following, we derive deterministic approximations to the frequency of a neutral allele linked to loci under purifying selection after a single pulse of admixture. We consider a neutral polymorphism on an autosome and on the X chromosome, and in both cases we allow for one or multiple linked loci under selection.

A single autosomal locus under selection

We model the allele-frequency dynamics at a neutral locus linked to a selected locus following a single pulse of admixture (introgression) from the Neanderthal population that happened t generations ago. Let N_1 and N_2 be the Neanderthal and human alleles at the neutral locus, and S_1 and S_2 the two alleles at the locus under selection. We denote the recombination rate between the two loci by r, and assume that the Neanderthal-derived allele S_1 is deleterious in humans. Specifically, the viability of a human individual heterozygous at the locus under selection is $w_{12} = w_{21} = 1 - s$, where $0 < s < 1$, and the viability of a human S_2 homozygote is $w_{22} = 1$. We further assume that the frequency of S_1 in humans is low, so that the deleterious

S_1S_1 homozygotes are very rare and can be ignored. Numerical and individual-based simulations (see below) show that this assumption is reasonable for the parameter values we consider. We assume that, prior to admixture, Neanderthals and humans were fixed for allele N_1 and N_2, respectively. At the time of introgression, the frequency of N_1 rises instantaneously from 0 to p_0 in the human population.

In the following, we describe how p_t, the frequency of N_1 t generations after introgression, depends on its initial frequency p_0, the heterozygote selection coefficient s, and the recombination rate r. Let x_0 and y_0 denote the frequency of haplotypes S_1N_1 and S_2N_1 in humans at the time of admixture. Hence, the frequency of allele N_1 immediately after admixture is

$$p_0 = x_0 + y_0. \tag{1}$$

In the following we assume that $x_0 + y_0 \ll 1$, and so we can ignore the effects of selection against homozygotes.

After recombination and random union of gametes, the haplotype frequencies in zygotes can be approximated by

$$x_0^* = x_0(1 - r), \tag{2a}$$

$$y_0^* = x_0 r + y_0, \tag{2b}$$

still assuming that S_1 is initially rare in humans.

After viability selection, the haplotype frequencies in the next generation of adult humans become

$$x_1 = x_0^*(1 - s) = x_0(1 - r)(1 - s), \tag{3a}$$

$$y_1 = y_0^* = x_0 r + y_0. \tag{3b}$$

From here, it is straightforward to obtain the explicit equations for the haplotype frequencies at generation t as

$$x_t = x_0[(1 - r)(1 - s)]^t, \tag{4a}$$

$$y_t = r \sum_{i=0}^{t-1} x_i + y_0. \tag{4b}$$

This can be simplified to

$$x_t = x_0[(1-r)(1-s)]^t, \tag{5a}$$

$$y_t = x_0 r \frac{1-[(1-s)(1-r)]^t}{1-(1-r)(1-s)} + y_0. \tag{5b}$$

Plugging equation (5) into equation (1), we obtain

$$p_t = x_0 f(r,s,t) + y_0, \tag{6}$$

where

$$f(r,s,t) = \frac{[(1-r)(1-s)]^t[1-r-(1-r)(1-s)]+r}{1-(1-r)(1-s)}. \tag{7}$$

As time t goes to infinity, $f(r,s,t)$ approaches Bengtsson's [1] gene flow factor for the case when selection happens before migration (cf. Eq. A3 in [2]):

$$p_\infty = x_0 \frac{r}{1-(1-r)(1-s)} + y_0. \tag{8}$$

If s and r are small, at equilibrium $f(r,s,t)$ further simplifies to $r/(r+s)$, which is equal to the approximation to the effective rate of gene flow found by Petry [3] based on a diffusion approximation. If $y_0 = 0$, we can replace x_0 by p_0 in the equations above.

A single X-chromosomal locus under selection

The non-pseudoautosomal X chromosome is a special case, because of the differences in transmission and the fact that recombination only happens in females. We take this into account by modifying Eq. (2) to

$$x^*_{\mathrm{X},0} = \frac{2}{3}x_{\mathrm{X},0}(1-r) + \left(1-\frac{2}{3}\right)x_{\mathrm{X},0} = x_{\mathrm{X},0}\left(1-\frac{2}{3}r\right), \tag{9a}$$

$$y^*_{\mathrm{X},0} = \frac{2}{3}x_{\mathrm{X},0}r + y_{\mathrm{X},0}. \tag{9b}$$

The factor of 2/3 accounts for the fact that two thirds of all X chromosomes are found in females.

As above, we assume that selection acts on viability and after recombination and random mating, but we now allow for selection to be sex specific. We denote by s_f and s_m the strength of selection against female and male carriers of a single S_1 allele, respectively. Recalling that, at the time of selection, a proportion of 2/3 of the X chromosomes are found in females, and 1/3

in males, we obtain the haplotype frequencies in adults of the next generation as

$$\begin{aligned} x_{\mathrm{X},1} &= \frac{2}{3} x^*_{\mathrm{X},0}(1-s_f) + \frac{1}{3} x^*_{\mathrm{X},0}(1-s_m) \\ &= x_{\mathrm{X},0}\left(1-\frac{2}{3}r\right)\left(1-\frac{1}{3}s_m-\frac{2}{3}s_f\right), \end{aligned} \tag{10a}$$

$$\begin{aligned} y_{\mathrm{X},1} &= y^*_{\mathrm{X},0} \\ &= \frac{2}{3} x_{\mathrm{X},0} r + y_{\mathrm{X},0}. \end{aligned} \tag{10b}$$

Iteration of Eq. (10) yields the explicit haplotype frequencies at time t,

$$x_{\mathrm{X},t} = x_{\mathrm{X},0}\left[\left(1-\frac{2}{3}r\right)\left(1-\frac{1}{3}s_m-\frac{2}{3}s_f\right)\right]^t, \tag{11a}$$

$$y_{\mathrm{X},t} = r x_{\mathrm{X},0}\frac{2}{3}\frac{1-\left[\left(1-\frac{2}{3}r\right)\left(1-\frac{1}{3}s_m-\frac{2}{3}s_f\right)\right]^t}{1-\left(1-\frac{2}{3}r\right)\left(1-\frac{1}{3}s_m-\frac{2}{3}s_f\right)} + y_{\mathrm{X},0}. \tag{11b}$$

The frequency of allele N_1 at time t, $p_{\mathrm{X},t} = x_{\mathrm{X},t} + y_{\mathrm{X},t}$, can therefore be written as

$$p_{\mathrm{X},t} = x_{\mathrm{X},0}\frac{\left(1-\frac{2}{3}r\right)^{t+1}\left(1-\frac{1}{3}s_m-\frac{2}{3}s_f\right)^t\left(\frac{1}{3}s_m+\frac{2}{3}s_f\right)+\frac{2}{3}r}{1-\left(1-\frac{2}{3}r\right)\left(1-\frac{1}{3}s_m-\frac{2}{3}s_f\right)} + y_{\mathrm{X},0}. \tag{12}$$

By setting $s_f = s_m = s$ and allowing recombination to happen in the entire population, i.e. by replacing 1/3 and 2/3 by 0 and 1, respectively, we recover Eq. (6), as expected. At equilibrium ($t = \infty$), Eq. (12) becomes

$$p_{\mathrm{X},\infty} = x_{\mathrm{X},0}\frac{\frac{2}{3}r}{1-\left(1-\frac{2}{3}r\right)\left(1-\frac{1}{3}s_m-\frac{2}{3}s_f\right)} + y_{\mathrm{X},0}, \tag{13}$$

which can be approximated as

$$\begin{aligned} p_{\mathrm{X},\infty} &\approx x_{\mathrm{X},0}\frac{\frac{2}{3}r}{\frac{1}{3}s_m+\frac{2}{3}s_f+\frac{2}{3}r} + y_{\mathrm{X},0} \\ &= x_{\mathrm{X},0}\left(1+\frac{\frac{1}{2}s_m+s_f}{r}\right)^{-1} + y_{\mathrm{X},0} \end{aligned} \tag{14}$$

if both selection and recombination are weak ($s_m, s_f, r \ll 1$).

Multiple autosomal loci under selection

In the following, we consider a neutral locus embedded in a suite of multiple autosomal loci under purifying selection. Due to the complexities of multilocus models, we make the following simplifications. First, we assume that, immediately before introgression, all deleterious alleles

were fixed in Neanderthals, but absent in humans. Second, we directly consider the situation where the frequency of the neutral Neanderthal-derived alleles has reached equilibrium in the human population (i.e. $t = \infty$). This can be justified if purifying selection is strong relative to the time since introgression, the admixture proportion, and the recombination rate. Third, we directly draw on the above-mentioned analogy between the surviving proportion of a cohort of introgressed alleles and the effective rate of gene flow at a neutral locus experiencing linkage-mediated selection (cf. Eq. 7 and subsequent text). We can therefore approximate the equilibrium frequency of the introgressed neutral allele N_1 by modifying the multilocus version of the effective migration rate in Eq. (24) of reference [4].

Specifically, let there be I and J loci under selection on the left- and right-hand side of the focal neutral site, and denote them by A_i and B_j, respectively. The equilibrium frequency of the introgressed neutral allele N_1 can then be approximated as

$$p_{\infty,IJ} \approx p_0 \left(1 + \frac{a_1}{r_{\mathsf{A}_1}}\right)^{-1} \times \prod_{i=2}^{I} \left(1 + \frac{a_i}{\sum_{k=1}^{i-1} a_k + r_{\mathsf{A}_i}}\right)^{-1} \times \left(1 + \frac{b_1}{r_{\mathsf{B}_1}}\right)^{-1} \times \prod_{j=2}^{J} \left(1 + \frac{b_j}{\sum_{k=1}^{j-1} b_k + r_{\mathsf{B}_j}}\right)^{-1}, \tag{15}$$

where a_i and b_i are the selection coefficients against the deleterious mutations at locus A_i and B_j, respectively, and r_{A_i} and r_{B_j} are the recombination fractions between the neutral locus and the respective locus under selection. Equation (15) assumes that both selection and recombination are weak.

If we set the selection coefficient at all loci under selection to the same value s, Eq. (15) simplifies to

$$p_{\infty,IJ} \approx p_0 \prod_{i=1}^{I} \left[1 + \frac{s}{s(i-1) + r_i}\right]^{-1} \prod_{j=1}^{J} \left[1 + \frac{s}{s(j-1) + r_j}\right]^{-1}, \tag{16}$$

where r_i and r_j are short cuts for r_{A_i} and r_{B_j}, respectively.

To assess the accuracy of Eq. (15), we derived discrete-time recursion equations for a model with one neutral and two selected loci. As before, we assumed that the admixture proportion is small, so that homozygous carriers of Neanderthal-derived alleles are very rare in the human population and the dynamics of the full diploid model can be approximated by considering a haploid model with four haplotypes. In addition, we assumed that the mean fitness of the human population was not affected by the few carriers of deleterious introgressed mutations. To simplify our notation, we denote the two loci under selection by A and B, and use A_1 (A_2) and B_1 (B_2) for

the deleterious (advantageous) alleles at locus A and B, respectively. We consider the following two configurations: one in which the neutral locus N is flanked by one locus under selection on each side (A–N–B), and another where the neutral locus is flanked by two selected loci on one side (A–B–N). We denote by r_{XY} the recombination rate between locus X and Y, where $r_{\mathsf{XY}} = r_{\mathsf{YX}}$, and we assume that recombination distances accumulate additively across loci.

For configuration A–N–B, the four focal haplotypes are $A_1N_1B_1$, $A_1N_1B_2$, $A_2N_1B_1$, and $A_2N_1B_2$. We denote their frequencies among adults of generation t after viability selection but before recombination and random mating by $x_1(t)$, $x_2(t)$, $x_3(t)$, and $x_4(t)$, respectively. After recombination, random mating, and viability selection, the haplotype frequencies among adults of the next generation are

$$\begin{aligned}
x_1(t+1) &= w_1\,(1-r_{\mathsf{AN}})\,(1-r_{\mathsf{BN}})\,x_1(t),\\
x_2(t+1) &= w_2\left[(1-r_{\mathsf{AN}})\,r_{\mathsf{BN}}x_1(t) + (1-r_{\mathsf{AN}}+r_{\mathsf{AN}}r_{\mathsf{BN}})\,x_2(t)\right],\\
x_3(t+1) &= w_3\left[r_{\mathsf{AN}}\,(1-r_{\mathsf{BN}})\,x_1(t) + (1-r_{\mathsf{BN}}+r_{\mathsf{AN}}r_{\mathsf{BN}})\,x_3(t)\right],\\
x_4(t+1) &= w_4\left[r_{\mathsf{AN}}r_{\mathsf{BN}}x_1(t) + r_{\mathsf{AN}}\,(1-r_{\mathsf{BN}})\,x_2(t)\right.\\
&\qquad\left.+\,(1-r_{\mathsf{AN}})\,r_{\mathsf{BN}}x_3(t) + x_4(t)\right],
\end{aligned} \tag{17}$$

where w_i denotes the relative fitness of haplotype i.

For configuration A–B–N, the four focal haplotypes are $A_1B_1N_1$, $A_1B_2N_1$, $A_2B_1N_1$, and $A_2B_2N_1$. In this case, the haplotype frequencies follow the following recursions:

$$\begin{aligned}
x_1(t+1) &= w_1\,(1-r_{\mathsf{AB}})\,(1-r_{\mathsf{BN}})\,x_1(t),\\
x_2(t+1) &= w_2\,(1-r_{\mathsf{AB}}+r_{\mathsf{AB}}r_{\mathsf{BN}})\,x_2(t),\\
x_3(t+1) &= w_3\left[r_{\mathsf{AB}}\,(1-r_{\mathsf{BN}})\,x_1(t) + (1-r_{\mathsf{BN}})\,x_3(t)\right],\\
x_4(t+1) &= w_4\left[r_{\mathsf{BN}}x_1(t) + r_{\mathsf{AB}}\,(1-r_{\mathsf{BN}})\,x_2(t) + r_{\mathsf{BN}}x_3(t) + x_4(t)\right].
\end{aligned} \tag{18}$$

For both configurations, the frequency of the introgressed neutral allele N_1 at time t can be approximated by

$$p_t = p(t) = \sum_{i=1}^{4} x_i(t), \tag{19}$$

where the $x_i(t)$ evolve according to Eq. (17) and (18), depending on the configuration.

We assume that fitness is additive across loci, and parametrize it as

$$\begin{aligned}
w_1 &= 1 - a - b, \\
w_2 &= 1 - a, \\
w_3 &= 1 - b, \\
w_4 &= 1,
\end{aligned} \tag{20}$$

where $0 \leq a, b \leq 1$.

We numerically iterated Eqs. (17) and (18) and computed p_t according to Eq. (19) at each step until an equilibrium was reached. Specifically, we terminated the process when the absolute difference between consecutive predicted allele frequencies p_t and p_{t+1} became smaller than 10^{-9}. We also iterated Eqs. (17) and (18) over a fixed number of $t = 2000$ generations and computed p_t. The approximation in Eq. (15) performs very well if the underlying assumptions are met and an equilibrium has been reached (S6 Fig). However, if an equilibrium has not been reached, the approximation in Eq. (15) should not be used (compare upward black triangles to respective black curves in S6 Fig). Moreover, if the assumption of recombination being weak relative to selection is violated, Eq. (15) tends to underestimate the actual equilibrium frequency of the neutral introgressed allele, as expected. This effect is particularly strong if genetic distances between consecutive linked loci under selection are highly asymmetric (compare blue triangles and respective blue curves in S6 Fig B for weak r_{AB}).

Multiple X-chromosomal loci under selection

Finally, we turn to the case of a neutral locus linked to multiple loci under selection on the X chromosome. Our results for the autosomes indicate that estimates obtained under the single- and multilocus models are in close agreement. Given that our estimates for the X chromosome under a single-locus model are in a similar region of parameter space, we do not fit this final, multilocus model to data from the X chromosome, but we include it here for completeness. Let there be I and J loci under selection on the left- and right-hand side of the focal neutral site, and denote them by A_i and B_j, respectively, where $i = 1, \ldots, I$ and $j = 1, \ldots, J$. Together, our Eq. (13) and Eq. (24) from reference [4] suggest that the equilibrium frequency of the introgressed

neutral allele N_1 may be approximated as

$$p_{\mathrm{X},\infty,IJ} \approx p_{\mathrm{X},0} \left(1 + \frac{\frac{1}{2}a_{1,m} + a_{1,f}}{r_{\mathsf{A}_1}}\right)^{-1} \times \prod_{i=2}^{I} \left[1 + \frac{\frac{1}{2}a_{i,m} + a_{i,f}}{\sum_{k=1}^{i-1}\left(\frac{1}{2}a_{k,m} + a_{k,f}\right) + r_{\mathsf{A}_i}}\right]^{-1} \times \left(1 + \frac{\frac{1}{2}b_{1,m} + b_{1,f}}{r_{\mathsf{B}_1}}\right)^{-1} \times \prod_{j=2}^{J} \left[1 + \frac{\frac{1}{2}b_{j,m} + b_{j,f}}{\sum_{k=1}^{j-1}\left(\frac{1}{2}b_{k,m} + b_{k,f}\right) + r_{\mathsf{B}_j}}\right]^{-1}, \tag{21}$$

where $a_{i,f}$ $(a_{i,m})$ and $b_{j,f}$ $(b_{j,m})$ are the coefficients of selection against heterozygous carriers of the deleterious mutation at locus A_i and B_j in females (males). Moreover, r_{A_i} and r_{B_j} are the recombination rates between the neutral locus and locus A_i and B_j, respectively. The factor of 1/2 accounts for the fact that a given X-chromosomal haplotype spends half of its time in males relative to the time spent in females.

If we fix the selection coefficients in females and males across all loci to s_f and s_m as above, Eq. (21) simplifies to

$$p_{\mathrm{X},\infty,IJ} \approx p_{\mathrm{X},0} \prod_{i=1}^{I} \left[1 + \frac{\frac{1}{2}s_m + s_f}{\left(\frac{1}{2}s_m + s_f\right)(i-1) + \frac{2}{3}r_i}\right]^{-1} \times \prod_{j=1}^{J} \left[1 + \frac{\frac{1}{2}s_m + s_f}{\left(\frac{1}{2}s_m + s_f\right)(j-1) + \frac{2}{3}r_j}\right]^{-1}, \tag{22}$$

where r_i and r_j are short cuts for r_{A_i} and r_{B_j}, respectively.

To test our conjecture in Eq. (21), we derived discrete-time recursions for a model with one neutral and two selected loci analogous to those in Eq. (17) and (18), but for the case of the X chromosome. As before, we assumed that the admixture proportion is small, so that the diploid model can be approximated by a haploid model with four haplotypes, and the mean fitness of the human population is not affected by introgression of deleterious mutations. We again simplify our notation by denoting the two loci under selection by A and B, and we use A_1 (A_2) and B_1 (B_2) for the deleterious (advantageous) alleles at locus A and B, respectively. As above, we consider the two configurations A–N–B and A–B–N, and assume that recombination rates accumulate additively across loci.

For configuration A–N–B, the four haplotypes of interest are $A_1N_1B_1$, $A_1N_1B_2$, $A_2N_1B_1$,

and $A_2N_1B_2$, and their frequencies after recombination in generation t are given by

$$\begin{aligned}
x_1^*(t) &= \frac{1}{3}x_1(t) + \frac{2}{3}\left(1 - r_{\mathsf{AN}}\right)\left(1 - r_{\mathsf{BN}}\right)x_1(t), \\
x_2^*(t) &= \frac{1}{3}x_2(t) + \frac{2}{3}\left[\left(1 - r_{\mathsf{AN}}\right)r_{\mathsf{BN}}x_1(t) + \left(1 - r_{\mathsf{AN}} + r_{\mathsf{AN}}r_{\mathsf{BN}}\right)x_2(t)\right], \\
x_3^*(t) &= \frac{1}{3}x_3(t) + \frac{2}{3}\left[r_{\mathsf{AN}}\left(1 - r_{\mathsf{BN}}\right)x_1(t) + \left(1 - r_{\mathsf{BN}} + r_{\mathsf{AN}}r_{\mathsf{BN}}\right)x_3(t)\right], \\
x_4^*(t) &= x_4(t) \\
&\quad + \frac{2}{3}\left[r_{\mathsf{AN}}r_{\mathsf{BN}}x_1(t) + r_{\mathsf{AN}}\left(1 - r_{\mathsf{BN}}\right)x_2(t) + \left(1 - r_{\mathsf{AN}}\right)r_{\mathsf{BN}}x_3(t)\right],
\end{aligned} \tag{23}$$

respectively. After random mating and viability selection, the frequency of haplotype i among adults in the next generation is

$$x_i(t+1) = \frac{1}{3}m_i x_i^*(t) + \frac{2}{3}f_i x_i^*(t), \tag{24}$$

where m_i and f_i are the relative fitnesses of haplotype i in males and females, respectively. The frequency of the introgressed neutral allele N_1 at time t is then obtained from Eq. (19), where the $x_i(t)$ behave as described in Eq. (24).

For configuration A–B–N, the four haplotypes of interest are $A_1B_1N_1$, $A_1B_2N_1$, $A_2B_1N_1$, and $A_2B_2N_1$. Their frequencies after recombination in generation t are

$$\begin{aligned}
x_1^*(t) &= \frac{1}{3}x_1(t) + \frac{2}{3}\left(1 - r_{\mathsf{AB}}\right)\left(1 - r_{\mathsf{BN}}\right)x_1(t), \\
x_2^*(t) &= \frac{1}{3}x_2(t) + \frac{2}{3}\left(1 - r_{\mathsf{AB}} + r_{\mathsf{AB}}r_{\mathsf{BN}}\right)x_2(t), \\
x_3^*(t) &= \frac{1}{3}x_3(t) + \frac{2}{3}\left[r_{\mathsf{AB}}\left(1 - r_{\mathsf{BN}}\right)x_1(t) + \left(1 - r_{\mathsf{BN}}\right)x_3(t)\right], \\
x_4^*(t) &= x_4(t) + \frac{2}{3}\left[r_{\mathsf{BN}}x_1(t) + r_{\mathsf{AB}}\left(1 - r_{\mathsf{BN}}\right)x_2(t) + r_{\mathsf{BN}}x_3(t)\right],
\end{aligned} \tag{25}$$

respectively. Note that $r_{\mathsf{AN}} = r_{\mathsf{AB}} + r_{\mathsf{BN}}$ by assumption. Equations (24) and (19) remain unchanged.

As above, we assume that fitness is additive across loci. For females, we parametrize fitnesses as

$$\begin{aligned}
f_1 &= 1 - a_f - b_f, \\
f_2 &= 1 - a_f, \\
f_3 &= 1 - b_f, \\
f_4 &= 1,
\end{aligned} \tag{26}$$

and for males, we set

$$\begin{aligned} m_1 &= 1 - a_m - b_m, \\ m_2 &= 1 - a_m, \\ m_3 &= 1 - b_m, \\ m_4 &= 1, \end{aligned} \tag{27}$$

where $0 \leq a_f, a_m, b_f, b_m \leq 1$.

We numerically iterated Eq. (24) and computed p_t according to Eq. (19) at each step until an equilibrium was reached. As above, we terminated the process when the absolute difference between consecutive allele frequencies p_t and p_{t+1} became smaller than 10^{-9}. We also iterated Eq. (24) over a fixed number of $t = 2000$ generations and computed p_t. As expected, our conjecture in Eq. (21) provides a very good approximation if the underlying assumptions are met and an equilibrium has been reached (S7 Fig). However, as in the autosomal case discussed above, if an equilibrium has not been reached, or if the assumption of recombination being weak relative to selection is violated, the approximation in Eq. (21) tends to underestimate the actual frequency of the neutral introgressed allele (S7 Fig).

Evaluating the accuracy of approximations

We evaluated the accuracy of Eqs. (6) and (12) in two ways. For each test we chose the range of values of r based on the size of genomic windows used in our analysis of the human data. We chose the range for s such that it includes the point estimate obtained from our inference procedure (see S2 Text for details).

First, we tested the effects of ignoring homozygous individuals by numerically iterating discrete-time recursions for a model with two loci under selection, after setting the selection coefficient at one of these to zero. For the case of autosomes, we used the recursions given in Eq. (2.9) on page 45 in reference [5] (cf. [6]). For the X chromosome, we used those from Eq. (10) in reference [7] after correcting them by substituting F for $2F$. We verified Eqs. (6) and (8), and Eqs. (12) and (13) by comparison to values obtained through iteration of the recursions for the autosomes and X chromosome, respectively. To obtain the equilibrium values using recursions, we iterated the latter until the difference in allele frequency between two generations was less than 10^{-9}. The results of this comparison suggest that ignoring homozygous individuals does not affect p_t substantially (S1 Fig–S4 Fig).

Our inference method (S2 Text) is based on the deterministic expressions for the Neanderthal

allele frequency derived above, and we view drift as 'noise' around these expectations. In a second test, we therefore assessed whether genetic drift has a substantial influence on the average frequency of the focal neutral allele. We did this by performing individual-based simulations and calculating the difference between the frequency of the neutral alleles after $t = 2000$ generations ($p_{t,\mathrm{sim}}$) and that obtained from Eq. (6) with $t = 2000$. For the individual-based simulations, we assumed a single constant population size of $N = 10,000$ diploid individuals of which $2Np_0$ are double heterozygous (N_2S_2/N_1S_1) at the start. Generations are non-overlapping. We modeled soft selection and assumed a multiplicative fitness scheme. This means that individuals homozygous for deleterious allele have fitness $(1-s)^2$. Plotting $p_{t,\mathrm{sim}}$ against p_t along with approximate 95% CI for $p_{t,\mathrm{sim}}$, we found a good agreement between average observed and expected deterministic results (S5 Fig). This shows that our approach of using deterministic equations is valid in our parameter regime.

In conclusion, our tests suggest that for the parameter range of interest, our approximations describe the expected frequency of neutral alleles well.

Models of waves of introgression

Hybridization with Neanderthals likely happened over many generations, rather than in one generation as we assumed above with the single-pulse admixture model. Furthermore, there is evidence of at least two waves of Neanderthal introgression into the East Asian population [8–11]. In this section, we derive expressions for the present-day frequency of a neutral Neanderthal allele linked to a single locus exposed to purifying selection under a number of more complicated models. Specifically, we start by considering how haplotype frequencies change over time during continuous introgression. We then use this result to derive the equivalent expressions for models with one and two discrete waves of introgression. Lastly, we show that the a single-pulse model cannot be distinguished from the wave models if the onset and duration of admixture periods are unknown.

Haplotype frequencies during continuous introgression

In the simplest model of continuous introgression, each generation a constant fraction of N_1S_1 and N_1S_2 haplotypes flow into the human population. We consider a slightly more complex model where the fraction of haplotypes that introgresses into humans at the initial admixture event is x_0 and y_0, but mx_0 and ny_0 for all future generations, where $m > 0$ and $n > 0$. This parametrization will be useful when we derive a model of two waves of introgression below.

The following events happen each generation: 1) admixture (inflow of Neanderthal alleles) at the adult stage, 2) recombination, mating, gamete production, random union of gametes, and 3) viability selection. During the initial admixture event, the frequency of N_1S_1 and N_1S_2 in humans rises instantaneously from 0 to x_0 and y_0, respectively. Recombination and viability selection then alter the haplotype frequencies according to Eq. (2) and (3), respectively. From the first generation onward, introgression increases the frequency of Neanderthal haplotypes in humans by mx_0 and ny_0. Therefore, for generations $t = 1, 2, 3, \ldots$, the haplotype frequencies after admixture become

$$\begin{aligned} x_t^\circ &= x_t + x_0 m, \\ y_t^\circ &= y_t + y_0 n. \end{aligned} \tag{28}$$

After recombination, we have

$$\begin{aligned} x_t^* &= x_t^\circ(1-r), \\ y_t^* &= x_t^\circ r + y_t^\circ, \end{aligned} \tag{29}$$

and after viability selection, the haplotype frequencies among adults in the next generation are

$$\begin{aligned} x_{t+1} &= x_t^*(1-s), \\ y_{t+1} &= y_t^*. \end{aligned} \tag{30}$$

Using the same methods as for the single-pulse model, we find the explicit expressions for the frequency of N_1S_1 and N_1S_2 in the human population in generation t as

$$\begin{aligned} x_t &= x_0 \left(G^t + m\frac{G^t - G}{G-1} \right), \\ y_t &= x_0 r \left[1 + \frac{G^t - G}{G-1} + m\frac{G^t - tG + t - 1}{(G-1)^2} \right] + y_0[1 + (t-1)n], \end{aligned} \tag{31}$$

where

$$G = (1-r)(1-s). \tag{32}$$

Single-wave introgression model

We now consider a model with a single wave of continuous introgression over τ generations, after which introgression stops. Therefore, during the first τ generations, haplotype frequencies change according to the model of continuous introgression discussed above (Eq. 31). For $t > \tau$, haplotype frequencies change according to the single-pulse model with initial frequencies x_τ and

y_τ (Eq. 5). Therefore we can write

$$x_t = \begin{cases} x_0 \left(G^t + m \frac{G^t - G}{G-1} \right) & \text{if } t \leq \tau, \\ x_\tau G^{t-\tau} & \text{if } \tau < t, \end{cases} \tag{33}$$

and

$$y_t = \begin{cases} x_0 r \left[1 + \frac{G^t - G}{G-1} + m \frac{G^t - tG + t - 1}{(G-1)^2} \right] + y_0 [1 + (t-1)n] & \text{if } t \leq \tau, \\ x_\tau r \left(\frac{1 - G^{t-\tau}}{1-G} \right) + y_\tau & \text{if } \tau < t, \end{cases} \tag{34}$$

where

$$\begin{aligned} x_\tau &= x_0 \left(G^\tau + m \frac{G^\tau - G}{G-1} \right), \\ y_\tau &= x_0 r \left[1 + \frac{G^\tau - G}{G-1} + m \frac{G^\tau - tG + \tau - 1}{(G-1)^2} \right] + y_0 [1 + (\tau - 1)n], \\ m &= n = 1. \end{aligned} \tag{35}$$

Dual-wave introgression model

It is straighforward to extend the model of a single wave of continuous introgression to include further waves. We do this here for a second wave. Specifically, introgression occurs during a first wave of length τ_1 generations, after which it stops until generation τ_2, when the second wave of introgression starts. The second wave ends at generation τ_3. Up until τ_2 this model is equivalent to the single-wave introgression model introduced above. Therefore, the expressions for x_t and y_t for $t \leq \tau_2$ are given by Eqs. (33) and (34), after replacing τ by τ_1. At generation τ_2, introgression starts again, but the initial haplotype frequencies are now x_{τ_2} and y_{τ_2}, rather than 0. This situation is equivalent to the continuous introgression model starting from $t = 1$ (rather than $t = 0$) and with x_0 and y_0 replaced by x_{τ_2} and x_{τ_2}.

Then, the expressions for x_t and y_t can be found to be

$$x_t = \begin{cases} x_0 \left(G^t + m \frac{G^t - G}{G-1} \right) & \text{if } t \leq \tau_1, \\ x_{\tau_1} G^{t-\tau_1} & \text{if } \tau_1 < t \leq \tau_2, \\ x_{\tau_2} \left(G^{t-\tau_2+1} + m_{\tau_2} \frac{G^{t-\tau_2+1} - G}{G-1} \right) & \text{if } \tau_2 < t \leq \tau_3, \\ x_{\tau_3} G^{t-\tau_3} & \text{if } \tau_3 < t, \end{cases} \tag{36}$$

and

$$y_t = \begin{cases} x_0 r\left[1+\frac{G^t-G}{G-1}+m\frac{G^t-tG+t-1}{(G-1)^2}\right]+y_0\left[1+(t-1)n\right] & \text{if } t \le \tau_1, \\ x_{\tau_1} r\frac{1-G^{t-\tau_1}}{1-G}+y_{\tau_1} & \text{if } \tau_1 < t \le \tau_2, \\ x_{\tau_2} r\left[1+\frac{G^{t-\tau_2+1}-G}{G-1}+m_{\tau_2}\frac{G^{t-\tau_2+1}-(t-\tau_2+1)G+(t-\tau_2+1)-1}{(G-1)^2}\right] & \\ \qquad +y_{\tau_2}\left[1+(t-\tau_2)n_{\tau_2}\right] & \text{if } \tau_2 < t \le \tau_3, \\ x_{\tau_3} r\frac{1-G^{t-\tau_3}}{1-G}+y_{\tau_3} & \text{if } \tau_3 < t, \end{cases} \tag{37}$$

where

$$\begin{aligned} x_{\tau_1} &= x_0\left(G^{\tau_1}+m\frac{G_1^{\tau}-G}{G-1}\right), \\ x_{\tau_2} &= x_{\tau_1}G^{\tau_2-\tau_1}, \\ x_{\tau_3} &= x_{\tau_2}\left(G^{\tau_3-\tau_2+1}+m_{\tau_2}\frac{G^{\tau_3-\tau_2+1}-G}{G-1}\right), \\ y_{\tau_1} &= x_0 r\left[1+\frac{G^{\tau_1}-G}{G-1}+m\frac{G^{\tau_1}-\tau_1 G+\tau_1-1}{(G-1)^2}\right]+y_0[1+(\tau_1-1)n], \\ y_{\tau_2} &= x_{\tau_1} r\left(\frac{1-G^{\tau_2-\tau_1}}{1-G}\right)+y_{\tau_1}, \\ y_{\tau_3} &= x_{\tau_2} r\left[1+\frac{G^{\tau_3-\tau_2+1}-G}{G-1}+m_{\tau_2}\frac{G^{\tau_3-\tau_2+1}-(t-\tau_2+1)G+(\tau_3-\tau_2+1)-1}{(G-1)^2}\right], \\ &+y_{\tau_2}[1+(\tau_3-\tau_2)n_{\tau_2}], \\ m &= n = 1, \\ m_{\tau_2} &= x_0/x_{\tau_2}, \\ n_{\tau_2} &= y_0/y_{\tau_2}. \end{aligned} \tag{38}$$

In both the single-pulse and wave models, haplotype frequencies change at the same rate once introgression stops, and this change is determined by r and s. The difference is that the haplotype frequencies at the time when introgression stops can vary under different models. However, if we do not know the duration of the waves of introgression or the time between them, we can always represent wave models as a single-pulse model in which the pulse happened more recently. To approximately map the single-wave model to the single-pulse model, we replace t by $t-[\tau-(\ln x_\tau-\ln x_0)/\ln G]$ in Eq. (5). To do the same for the dual-wave model, we replace t by $t-[\tau_3-(\ln x_{\tau_3}-\ln x_0)/\ln G]$. The effect of this approximation is that after the time of the last introgression, the single-pulse and wave models are indistinguishable (S8 Fig). That is, we have shown that, if one does not know the details of introgression, it is impossible to distinguish between the single-pulse and wave models based only on the present-day frequency of

Neanderthal alleles. Therefore, the single-pulse model is a good model for our analysis, despite the fact that it is obviously an approximation.

References

[1] Bengtsson BO. The flow of genes through a genetic barrier. In: Greenwood PJ, Harvey PH, Slatkin M, editors. Evolution – Essays in honour of John Maynard Smith. vol. 1. New York, NY: Cambridge University Press; 1985. p. 31–42.

[2] Charlesworth B, Nordborg M, Charlesworth D. The effects of local selection, balanced polymorphism and background selection on equilibrium patterns of genetic diversity in subdivided populations. Genet Res. 1997 Oct;70(2):155–174.

[3] Petry D. The effect on neutral gene flow of selection at a linked locus. Theor Popul Biol. 1983;23(3):300–313.

[4] Aeschbacher S, Bürger R. The effect of linkage on establishment and survival of locally beneficial mutations. Genetics. 2014 May;197(1):317–336.

[5] Rice SH. Evolutionary Theory: Mathematical and Conceptual Foundations. Sunderland, MA: Sinauer Associates Inc; 2004.

[6] Lewontin RC, Kojima K. The evolutionary dynamics of complex polymorphisms. Evolution. 1960 Dec;14(4):458–472.

[7] Connallon T, Clark AG. Antagonistic versus nonantagonistic models of balancing selection: characterizing the relative timescales and hitchhiking effects of partial selective sweeps. Evolution. 2013;67(3):908–917.

[8] Wall JD, Yang MA, Jay F, Kim SK, Durand EY, Stevison LS, et al. Higher levels of neanderthal ancestry in East Asians than in Europeans. Genetics. 2013 May;194(1):199–209.

[9] Vernot B, Akey JM. Resurrecting Surviving Neandertal Lineages from Modern Human Genomes. Science. 2014 Feb 28;343(6174):1017–1021.

[10] Vernot B, Akey JM. Complex history of admixture between modern humans and Neandertals. Am J Hum Genet. 2015 Mar;96(3):448–453.

[11] Kim BY, Lohmueller KE. Selection and reduced population size cannot explain higher amounts of Neandertal ancestry in East Asian than in European human populations. Am J Hum Genet. 2015 Mar;96(3):454–461.

S1 Fig

Approximate frequency p_t of N_1 as a function of the recombinational distance r. Lines represent Eq. (6) for $t = 2000$ (red) and the equilibrium given in Eq. (8) (grey). Numerical iterations of the corresponding recursion equations are represented by red upward and black downward facing triangles. Other parameters are $s = 0.0001$, and $y_0 = 0$ for all lines, and $p_0 = 0.04$ (dotted), 0.034 (dashed) and 0.03 (full line).

S2 Fig

Approximate frequency p_t of N_1 as a function of the recombinational distance r. Lines represent Eq. (6) for $t = 2000$ (red) and the equilibrium given in Eq. (8) (grey). Numerical iterations of the corresponding recursion equations are represented by red upward and black downward facing triangles. Other parameters are $s = 0.0004$, and $y_0 = 0$ for all lines, and $p_0 = 0.04$ (dotted), 0.034 (dashed) and 0.03 (full line).

S3 Fig

Approximate frequency p_t of N_1 as a function of the recombinational distance r for the X chromosome. Lines represent Eq. (12) for $t = 2000$ (red) and the equilibrium from Eq. (13) (grey). Numerical iterations of the corresponding recursion equations are represented by red upward and black downward facing triangles. Other parameters are $s_f = s_m = 0.0001$, and $y_{X,0} = 0$ for all lines, and $p_0 = 0.04$ (dotted), 0.034 (dashed) and 0.03 (full line).

S4 Fig

Approximate frequency p_t of N_1 as a function of the recombinational distance r for the X chromosome. Lines represent Eq. (12) for $t = 2000$ (red) and the equilibrium from Eq. (13) (grey). Numerical iterations of the corresponding recursion equations are represented by red upward and black downward facing triangles. Other parameters are $s_f = s_m = 0.0004$, and $y_{X,0} = 0$ for all lines, and $p_0 = 0.04$ (dotted), 0.034 (dashed) and 0.03 (full line).

S5 Fig

Comparison of the mean frequency of N_1 obtained from individual-based simulations to the theoretical prediction from Eq. (6)**.** The figure shows 676 circles representing different combinations of r (recombination rate) and s (selection coefficient). Values of r range from 1×10^{-5} (red circle border) to 1×10^{-2} (black border), s ranges from 1×10^{-5} (yellow circle

area) to 4×10^{-4} (light blue area). For each parameter combination, the mean frequency of N_1 after $t = 2000$ generations was calculated across 1000 independent runs. Grey lines represent approximate 95% confidence intervals for simulation results (mean $\pm 1.96 \times$ standard error), and a black line with slope 1 is shown for reference.

S6 Fig

Accuracy of approximation to the frequency of a neutral allele N_1 linked to multiple autosomal loci under purifying selection. Curves show $p_{\infty,IJ}$ from Eq. (15) for various recombination distances between the focal neutral locus N and the two loci under selection, A and B. Upward and downward facing triangles give values obtained after iterating deterministic recursions over $t = 2000$ generations and until the equilibrium is reached, respectively. A: The neutral locus is flanked by one locus under selection on each side, and recursions followed Eq. (17). B: The neutral locus is flanked by two selected loci on one side and recursions followed Eq. (18). A, B: Selection coefficients against introgressed deleterious mutations at locus A and B are $a = 0.0002$ and $b = 0.0004$, respectively. The initial frequency of N_1 is $p_0 = 0.04$.

S7 Fig

Accuracy of approximation to the frequency of a neutral allele N_1 linked to multiple X-chromosomal loci under purifying selection. Curves show $p_{\mathrm{X},\infty,IJ}$ from Eq. (21) for various recombination distances between the focal neutral locus N and the two loci under selection, A and B. Upward and downward facing triangles give values obtained after iterating Eq. (24) over $t = 2000$ generations and until the equilibrium is reached, respectively. A, B: The neutral locus is flanked by one locus under selection on each side. C, D: The neutral locus is flanked by two loci under selection on one side. A, C: Selection coefficients against introgressed deleterious mutations at locus A and B in females (males) are $a_f = 0.0001$ ($a_m = 0.0003$) and $b_f = 0.0002$ ($b_m = 0.0006$), respectively. B, D: Selection coefficients are identical in the two sexes; $a_f = a_m = 0.0001$ and $b_f = b_m = 0.0002$. In all panels, the initial frequency of N_1 is $p_{\mathrm{X},0} = 0.04$.

S8 Fig

Mapping models with one (red line) and two (blue line) waves of introgression to a single-pulse model. By changing time in the single-pulse model (dashed and dotted black lines) as described in S1 Text, we can recover present-day haplotype frequencies generated by the

wave models. Parameters are $r = 10^{-4}$, $s = 5 \times 10^{-4}$, $x_0 = 0.04$, and $y_0 = 0.001$. The duration of admixture in the single-wave model is $\tau = 500$. Additional parameters for the dual-wave model are $\tau_1 = 75$, $\tau_2 = 1075$, $\tau_3 = 1500$. The solid black line represents a single-pulse model without change of time.
