# Supplementary material for: The Strength of Selection against Neanderthal Introgression
Source: PLoS Genet. 2016 Nov 8;12(11):e1006340. doi: 10.1371/journal.pgen.1006340 (PMC5100956; doi:10.1371/journal.pgen.1006340)
Supplement: S2 Text — Here, we introduce the last model parameter, the average probability μ that, at any given exonic base pair, a deleterious Neanderthal allele is segregating in the modern human population. We then discuss the details of our inference procedure and expand on our results. (PDF) [file pgen.1006340.s002.pdf]

S2 Text

Inference Procedure

Ivan Juric, Simon Aeschbacher, Graham Coop

In S1 Text we describe how the present-day frequency of a neutral Neanderthal allele depends on the selection coefficient s, the recombinational distance(s) from the (neareast-neighbouring) site(s) under purifying selection, and the initial frequency p_0 of the Neanderthal allele. Here, we introduce the last model parameter, the average probability μ that, at any given exonic base pair, a deleterious Neanderthal allele is segregating in the modern human population. We then discuss the details of our inference procedure and expand on our results.

Theory

Incorporating the exonic density of sites under purifying selection

Some loci are more likely to harbor deleterious alleles than others. For example, mutations at exonic sites are more likely to affect fitness than mutations in introns or in non-genic regions. In our models, we assume that any given exonic site in the human population may harbor a deleterious Neanderthal-derived allele with a certain probability. We denote this probability by μ and assume that it is equal for all exonic sites. In this sense, μ also denotes the exonic density of deleterious Neanderthal alleles in modern humans.

We start by considering a focal neutral locus ℓ on an autosome, surrounded by $\mathcal{J}_\ell$ exonic sites contained in a window of a given genetic length. We then order the exonic sites by their absolute physical distance from ℓ, and denote by r_j the recombination rate between the jth exonic site and ℓ ($j = 1, 2, 3, \ldots, \mathcal{J}_\ell$). If the exonic density μ of sites under purifying selection is low, any focal neutral site ℓ will on average be linked to at most one deleterious site. Therefore, we can approximate the expected frequency of the neutral Neanderthal allele at locus ℓ by considering only the effect of the nearest-neighboring exonic site under selection. In this case, the probability that the jth exonic site is the nearest-neighbouring deleterious site is given by $\mu(1-\mu)^{j-1}$, and the frequency $p_{\ell,t}$ of the neutral Neanderthal allele at locus ℓ at time t is given by $x_0 f(r_j, s, t) + y_0$

(Eq. 6 in S1 Text).

Summing over all $\mathcal{J}_\ell$ exonic sites, and accounting for the case where none of them is under selection, we obtain the expectation of $p_{\ell,t}$ with respect to its genomic window as

$$\begin{aligned} \mathrm{E}[p_{\ell,t}] &= \sum_{j=1}^{\mathcal{J}_\ell} \mu(1-\mu)^{j-1}\left[x_0 f(r_j,s,t)+y_0\right] + (1-\mu)^{\mathcal{J}_\ell}(x_0+y_0) \\ &= x_0\left[\mu\sum_{j=1}^{\mathcal{J}_\ell}(1-\mu)^{j-1} f(r_j,s,t) + (1-\mu)^{\mathcal{J}_\ell}\right] + y_0. \end{aligned} \tag{1}$$

To obtain the expression for the expected Neanderthal allele frequency on the X chromosome, we replace x_0, y_0 and $f(r_j,s,t)$ in Eq. (1) above by terms corresponding to the X chromosome (cf. Eq. 12 in S1 Text). In practice, we infer μ to be small, which allows us to estimate $\mathrm{E}[p_{\ell,t}]$ by summing over exons rather than all exonic sites. This approximation substantially increases computational efficiency, and leads to Eq. (9), described later in this supplement.

Inference procedure

Residual sum of squared differences (RSS)

Our inference method relies on finding the parameters that minimize, across all SNPs, the residual sum of squared differences (RSS) between the observed Neanderthal allele frequency p_n from reference [1], and the one expected in a present-day human sample, $\mathrm{E}[p_{\ell,t}]$ (see S1 Text for a derivation). Specifically, the RSS is given by

$$\mathrm{RSS} = \sum_{\ell=1}^{L}(p_{\ell,n} - \mathrm{E}[p_{\ell,t}])^2 = \sum_{\ell=1}^{L}\left[p_{\ell,n} - x_0 g_\ell(\mathbf{r},s,t,\mu) - y_0\right]^2, \tag{2}$$

where the sum is over all L SNPs in the autosomal genome (or on the X chromosome). We can rewrite Eq. (2) as

$$\mathrm{RSS} = \sum_{\ell=1}^{L} p_{\ell,n}^2 - 2x_0\sum_{\ell=1}^{L} p_{\ell,n} g_\ell - 2y_0\sum_{\ell=1}^{L} p_{\ell,n} + x_0^2\sum_{\ell=1}^{L} g_\ell^2 + 2x_0y_0\sum_{\ell=1}^{L} g_\ell + y_0^2 L, \tag{3}$$

where, for clarity, we write g_ℓ instead of $g_\ell(\mathbf{r},s,t,\mu)$. When $y_0 = 0$, i.e. if we assume that deleterious alleles are fixed in Neanderthals, Eq. (3) simplifies to

$$\mathrm{RSS} = \sum_{\ell=1}^{L} p_{\ell,n}^2 - 2p_0\sum_{\ell=1}^{L} g_\ell p_{\ell,n} + p_0^2\sum_{\ell=1}^{L} g_\ell^2. \tag{4}$$

Minimizing the RSS

For given values of μ and s, all of the sums in Eq. (3) are constants, and the minimum RSS depends only on x_0 and y_0. This function, which we will refer to as $\mathrm{RSS}(x_0, y_0)$, has a minimum if $D(x_c, y_c) > 0$ and $\mathrm{RSS}_{xx}(x_c, y_c) > 0$, where

$$D(x_c, y_c) = \mathrm{RSS}_{xx}(x_c, y_c)\mathrm{RSS}_{yy}(x_c, y_c) - \left[\mathrm{RSS}_{xy}(x_c, y_c)\right]^2, \tag{5}$$

and $\mathrm{RSS}_{xx}(x_c, y_c)$, $\mathrm{RSS}_{yy}(x_c, y_c)$, and $\mathrm{RSS}_{xy}(x_c, y_c)$ are the second-order partial derivatives of $\mathrm{RSS}(x_0, y_0)$ with respect to x_0 and y_0, and the cross partial derivative with respect to x_0 and y_0, respectively, all evaluated at the critical points x_c, y_c. We find the critical points by solving a system of two equations obtained by setting the first-order derivatives to zero: $\mathrm{RSS}_x(x_0, y_0) = 0$ and $\mathrm{RSS}_y(x_0, y_0) = 0$. We find

$$x_c = \frac{\sum p_{\ell,n} \sum g_\ell - L \sum p_{\ell,n} g_\ell}{\left(\sum g_\ell\right)^2 - L \sum g_\ell^2}, \tag{6a}$$

$$y_c = \frac{\sum p_{\ell,n} g_\ell \sum g_\ell - \sum p_{\ell,n} \sum g_\ell^2}{\left(\sum g_\ell\right)^2 - L \sum g_\ell^2}. \tag{6b}$$

Moreover,

$$\mathrm{RSS}_{xx}(x_c, y_c) = 2 \sum g_\ell^2, \tag{7a}$$

$$D(x_c, y_c) = 4 \left[L \sum g_\ell^2 - \left(\sum g_\ell\right)^2\right], \tag{7b}$$

where we have dropped indices in the summations for clarity. By the Cauchy–Schwarz inequality, $D(x_c, y_c)$ is always positive. The derivative $\mathrm{RSS}_{xx}(x_c, y_c)$ is also positive since $g_\ell > 0$. Therefore, x_c and y_c minimize the RSS for given values of μ and s.

We note that, technically, x_c and y_c can be less than zero. A negative value of x_c occurs if $\sum p_{\ell,n} \left(\sum g_\ell - L g_\ell\right) > 0$ while y_c is negative if $\sum p_{\ell,n} \left(g_\ell \sum g_\ell - \sum g^2\right) > 0$. In reality, the initial frequencies of N_1S_1 (x_0) and N_1S_2 (y_0) are non-negative. Therefore, whenever the theoretical x_c or x_c are negative, the respective effective (biologically permissible) minimising value is zero. Specifically, if $x_c < 0$, the effective point estimate of x_0 is zero, and all N_1 alleles in the human population are linked to the non-deleterious allele S_2, and the point estimate of the initial frequency y_0 is given by the average present-day frequency of N_1. On the other hand, if $y_c < 0$, the effective point estimate of y_0 is zero, and Eq. (3) turns into Eq. (4), which can then be minimized with respect to x_0. Having accounted for the cases of negative x_c or y_c, we see that x_c and y_c are the coordinates that minimize $\mathrm{RSS}(x_0, y_0)$ given μ and μ.

For the model that only considers the nearest-neighboring exonic site under selection as described in S1 Text, we evaluate the RSS for particular values of μ and s, denoted by μ_i and s_j, as follows:

1. Calculate x_c and y_c using Eq. (6) given μ_i and s_j.

2. If $x_c < 0$, set x_0 to zero and y_c to the mean observed Neanderthal frequency, $\bar{p}_n$.

3. If $y_c < 0$, set y_0 to zero and x_c to $\sum p_{\ell,n} g_\ell / \sum g_\ell^2$.

4. Calculate the RSS using Eq. (3) by replacing x_0 and y_0 by x_c and y_c, respectively.

We repeat steps 1–4 for all combinations of i and j, and then obtain the point estimates of μ and s as the pair of μ_i and s_j that minimises the RSS over all combinations of tested values.

Technical implementation

A recent study pubished estimates of the frequency p_n of Neanderthal ancestry in modern-day Europeans (EUR) and East Asians (ASN) at numerous SNPs in the genome [1]. We downloaded those estimates, as well as physical and genetic positions of SNPs from the Reich Lab website. The genetic resolution of this map is 1×10^{-3} cM, so if two loci are closer than that distance, they are assigned the same position. We downloaded a list of exons from the UCSC Genome Bioinformatics browser assembly (hg19) to match the files containing p_n.

For genes with alternative splicing, we collapsed all overlapping exons to create exonic regions, each of which starts at the beginning of the left-most (i.e upstream) overlapping exon and ends at the end of the right-most (downstream) overlapping exon. For genes without alternative splicing, our exonic regions are equivalent to exons. For simplicity, we refer to exonic regions as exons.

For each focal neutral site ℓ, we considered only exons whose midpoint is within a 1 cM window from the focal neutral locus, i.e. 0.5 cM on each side (see main text for a justification). Alternatively, we also estimated parameters by minimizing the RSS after averaging both observed and expected allele frequencies across non-overlapping blocks of 0.1 cM. Parameters estimates using this proceedure agreed well with the results based on the approach described above, where each SNP is considered independently. This suggests that our choice of procedure to minimize the RSS on a per-SNP level did not affect our results much.

We used linear interpolation to determine the genetic position of the midpoint of each exon. For an exon starting at physical position x_1 and ending at position x_2, we first identified its

physical midpoint x_m, and then calculated its genetc position as

$$r_m = r_1 + (r_2 - r_1)\frac{x_m - y_1}{y_2 - y_1}, \tag{8}$$

where r_1 and r_2 are the genetic map positions of the closest SNP to the left (upstream) and right (downstream) of x_m, and y_1 and y_2 are the respective physical positions. For exons that are positioned left of the first (most upstream) SNP, we assumed that the recombination rate per base pair between such exons and the most downstream SNP is the same as between the first and second SNP. We dealt analogously with exons starting further downstream than the right-most SNP. In cases where the first (most downstream) SNP is assigned a genetic position of 0 by the genetic map, but were exons left of that SNP are known, we removed that SNP and assigned these exons a genetic position of 0.

Lastly, if μ is small, the probability that an exon of length l base pairs contains the selected site is approximately μl. We used this approximation to speed up the calculation of p_t. Most exons are short ($\mu l \ll 1$). However, for longer exons ($\mu l \approx 1$), we divided them repeatedly in half until the condition $\mu l \ll 1$ was satisfied. In the end, for a given SNP ℓ, we approximated Eq. (1) by

$$E[p_{\ell,t}] = x_0 \left[\sum_{i=1}^{\mathcal{I}_\ell} \mu l_i \prod_{j=1}^{i-1} (1 - \mu l_j) f(r_i, s, t) + \prod_{j=1}^{\mathcal{I}_\ell} (1 - \mu l_j) \right] + y_0, \tag{9}$$

where $\mathcal{I}_\ell$ is the number of exons whose midpoints are within a 1 cM window surrounding SNP ℓ. When $y_0 = 0$, i.e. the deleterious allele is fixed in Neanderthals, then $x_0 = p_0$, and

$$E[p_{\ell,t}] = p_0 \left[\sum_{i=1}^{\mathcal{I}_\ell} \mu l_i \prod_{j=1}^{i-1} (1 - \mu l_j) f(r_i, s, t) + \prod_{j=1}^{\mathcal{I}_\ell} (1 - \mu l_j) \right], \tag{10}$$

which leads to Eq. (9) in the main text.

To calculate the RSS for the multiple locus model, at each of the 676 (26^2) combinations of μ and s, we drew 30 replicates of n selected sites for each chromosome, and distributed those sites randomly across the exonic sites. The number of selected sites per chromosome, n, was drawn from a binomial distribution with parameters μ and n_{tot}, where n_{tot} is the total number of exonic sites on the chromosome. Then, for each SNP, we calculated p_t according to Eq. (16) from S1 Text, and took the mean of this across our 30 replicates. This average p_t at each SNP was then used to calculate the RSS.

To assess how sensitive our estimates are to the size of the window considered around each neutral site, we repeated our analyses with windows of size 10 cM (instead of 1 cM as above).

This did not change our estimates substantially for the multi-locus equilibrium model (see next section), but it increased the computation time substantially.

Results

Model fit

In and we report point estimates and 95% block bootstrap confidence intervals for parameters that minimize the RSS under different models. Our bootstrap method is explained in the methods section of the main text. In S9 Fig to S13 Fig we show the RSS surfaces for μ and s for different models, while S14 Fig shows the RSS surfaces for the initial frequency of the Neanderthal allele on the X chromosome, $p_{\mathrm{X},0}$.

In S15 Fig to S17 Fig we show the fit between the average observed frequency of Neanderthal alleles, binned by gene density per map unit, and the allele frequency predicted by our model. Each plot is created by first splitting the genome into segments of constant size (in cM), then counting the number of exonic sites in each segment and lastly binning segments into bins of equal size. Pearson correlation coefficients between observed and model-predicted average Neanderthal allele frequencies across all bins are given in for a range of bin sizes. For each bin, we calculate the average observed frequency of Neanderthal alleles and the frequency predicted by our model using parameters from .

To explore the effect of the size of the genomic window considered around each focal neutral site, we repeated the multilocus inference procedure with windows of length 10cM instead of 1 cM, for both the X chromosome and the autosomes. The point estimates for the parameters are shown in . In S18 Fig and S19 Fig we show the resulting RSS surfaces for the autosomes and for the X chromosome, respectively.

Throughout this work, we have made use of posterior mean calls of Neanderthal ancestry in modern humans as reported by a previous study [1]. To explore the sensitivity of our method to how Neanderthal alleles are called, we alternatively fit our model to a more stringent set of Neanderthal alleles. Specifically, we restricted our analysis to the high-confidence Neanderthal calls reported in ref. [1], where an allele in modern humans was considered to originate from Neanderthals if it had a marginal probability larger than 90% of being derived from Neanderthal ancestry. We used the frequency of these high-confidence calls at each site in humans as our set of p_ℓ for the autosomes. The resulting point estimates are given in , and the RSS surfaces are shown in S20 Fig. Our estimates of p_0 are necessarily lower if we rely only on the high-confidence calls, because a smaller fraction of EUR and ASN alleles can be confidently called Neanderthal at

any given site, as compared to the posterior average call. For both EUR and ASN populations, the RSS surfaces for s and μ suggest that parameter combinations with high values of both s and μ can be ruled out (S20 Fig). For the ASN sample the point estimates suggest a high s and low μ, while for EUR the point estimate of s is lower than for the full data set. We also note that these surfaces have wider ridges (suggesting an inverse relationship between s and μ), perhaps reflecting the higher degree of statistical noise associated with using only the high-confidence calls of Neanderthal ancestry. Overall, this analysis shows that our main conclusion that the observed levels of Neanderthal ancestry in modern humans can be understood as resulting from weak selection is not sensitive to the details of how Neanderthal ancestry was called.

Population	Scenario	p_0	s	μ
EUR	Single Locus, $t = \infty$	0.0315	1.02×10^{-4}	7.8×10^{-5}
EUR	Multiple Loci, $t = \infty$	0.0312	2.5×10^{-5}	1.2×10^{-4}
EUR	Multiple Loci (10 cM), $t = \infty$	0.0349	4.6×10^{-5}	8.1×10^{-5}
EUR	Single Locus, $t = 2000$	0.0338	4.12×10^{-4}	8.1×10^{-5}
EUR (high conf. calls)	Single Locus, $t = 200$	0.0120	2×10^{-4}	4.0×10^{-5}
EUR	Single Locus, $t = 2000$, X chr	0.0292	9.6×10^{-4}	8.1×10^{-5}
ASN	Single Locus, $t = \infty$	0.0349	8.8×10^{-5}	6.8×10^{-5}
ASN	Multiple Loci $t = \infty$	0.0350	3.7×10^{-5}	1.0×10^{-4}
ASN	Multiple Loci (10 cM) $t = \infty$	0.0377	3.6×10^{-5}	8.1×10^{-5}
ASN	Single Locus, $t = 2000$	0.0360	3.52×10^{-4}	6.9×10^{-5}
ASN (high conf. calls)	Single Locus, $t = 200$	0.0126	9×10^{-4}	1.0×10^{-7}
ASN	Single Locus, $t = 2000$, X chr	0.0298	1.6×10^{-4}	6.8×10^{-4}

A Table. Minimum RSS parameters for μ, s and p_0 for different models described in S1 Text. Figure 1 in the main text shows an example of $E[p_t]$ for single locus model, $t = 2000$, for part of chromosome 1.

Population	Scenario	p_0	s	μ
EUR	Single Locus, $t = \infty$	$[3.00, 3.30] \times 10^{-2}$	$[0.8, 1.4] \times 10^{-4}$	$[7.8, 8.1] \times 10^{-5}$
EUR	Single Locus, $t = 2000$	$[3.22, 3.52] \times 10^{-2}$	$[3.4, 5.2] \times 10^{-4}$	$[0.41, 1.2] \times 10^{-4}$
EUR	Single Locus, $t = 2000$, X chr	$[2.32, 3.53] \times 10^{-2}$	$[0.64, 2.08] \times 10^{-3}$	$[0.41, 1.6] \times 10^{-4}$
ASN	Single Locus, $t = \infty$	$[3.35, 3.66] \times 10^{-2}$	$[0.40, 1.6] \times 10^{-4}$	$[4.1, 8.1] \times 10^{-5}$
ASN	Single Locus, $t = 2000$	$[3.45, 3.86] \times 10^{-2}$	$[2.6, 5.4] \times 10^{-4}$	$[0.41, 1.6] \times 10^{-4}$
ASN	Single Locus, t = 2000, X chr	$[2.36, 3.9] \times 10^{-2}$	$[0, 4] \times 10^{-3}$	$[0.001, 1] \times 10^{-3}$

B Table. The 95% bootstrap confidence intervals for μ, s, and p_0 for different models.

Segment size	ρ_{EUR}	ρ_{ASN}
0.5 cM	0.871	0.881
1 cM	0.897	0.710
1.5 cM	0.887	0.546
2 cM	0.847	0.633

C Table. Correlation between the estimated and the observed mean Neanderthal allele frequency for bins created using segments of different sizes.

The X chromosome and sex-bias during admixture

In this subsection we describe how we estimated the sex bias during admixture. We say that admixture is biased towards Neanderthal males if significantly more than 50% of introgressed alleles came from male Neanderthals. Conversely, if significantly less than 50% of introgressed alleles came from male Neanderthals, we say that admixture was biased towards Neanderthal females. Otherwise, we say that admixture showed no sex bias. Consider a single generation of matings between humans and Neanderthals. Let m_1 be the frequency of Neanderthal male $\times$ human female matings and let m_2 be the frequency of Neanderthal female $\times$ human male matings. Further, let p_0 and $p_{\mathrm{X},0}$ be the initial frequency of Neanderthal autosomal and X-linked alleles. Then, based on Mendelian inheritance,

$$p_0 = \frac{1}{2}m_1 + \frac{1}{2}m_2, \tag{11a}$$
$$p_{\mathrm{X},0} = \frac{1}{3}m_1 + \frac{2}{3}m_2. \tag{11b}$$

Solving Eq. (11) for m_1 and m_2 yields

$$m_1 = 4p_0 - 3p_{\mathrm{X},0}, \tag{12a}$$
$$m_2 = 3p_{\mathrm{X},0} - 2p_0. \tag{12b}$$

Based on our estimates of p_0 and p_{X} () for the EUR population, we obtain $m_{1,\mathrm{EUR}} = 0.0476$, $m_{2,\mathrm{EUR}} = 0.02$, $m_{1,\mathrm{EUR}}/m_{2,\mathrm{EUR}} = 2.38$, and for the ASN population, we obtain $m_{1,\mathrm{ASN}} = 0.0546$, $m_{2,\mathrm{ASN}} = 0.0174$, $m_{1,\mathrm{ASN}}/m_{2,\mathrm{ASN}} = 3.14$. We note that the CI intervals for the ratios of estimates of m_1 to m_2 are wide and include unity. However, in the main text (see also Fig 4) we discuss that $\mu_{\mathrm{X}} s_{\mathrm{X}}$ and $p_{\mathrm{X},0}$ are confounded, so it is possible that mating was sex-biased if selection was a lot stronger on the X than on autosomes. However, it seems likely that both selection and sex-biased mating may have contributed to shaping X-to-autosome levels of admixture.

Weigthed least squares fitting

Using the simulations described in S1 Text, we calculated the variance of the frequencies of neutral alleles as a function of the respective mean frequencies. This relationship, shown in S21 Fig, is approximately linear, with zero intercept. A linear relationship is expected as the binomial variance due to genetic drift around the expectation is proportional to $p_t(1 - p_t)$, and this is approximately equal to p_t if p_t is small.

In our main analysis, we fit the level of Neanderthal introgression along the genome to our expectation, $\mathrm{E}(p_{\ell,t})$ using a least squares approach. In doing this, we assume that the variance of $p_{\ell,t}$ is constant. However, as mentioned above, our simulations suggest that under our model the variance of $p_{\ell,t}$ is approximately a linear function of $\mathrm{E}[p_{\ell,t}]$ (see S21 Fig). We can apply this to observed allele frequencies and write $\mathrm{Var}[p_{\ell,n}] = Cp_0 g_\ell(\mathbf{r}, s, t, \mu)$, where C is a constant. To assess the effect of unequal variance across the parameter range we also performed a weighted least-squares (WLS) anaylsis. Instead of RSS (Eq. 6 in the main text) we now minimize

$$\mathrm{WSS} = \sum_{\ell=1}^{n_l} w_\ell (p_{\ell,n} - \mathrm{E}[p_{\ell,t}])^2 = \sum_{\ell=1}^{n_l} w_\ell \left[p_{\ell,n} - p_0 g_\ell(\mathbf{r}, s, t, \mu)\right]^2, \quad (13)$$

where w_ℓ are weights pertaining to each focal neutral site ℓ. We set the weights to be inversely proportional to the variance. Specifically, $w_\ell = 1/(Cp_0 g_\ell)$, and the equation to be minimized becomes

$$\mathrm{WSS} = \frac{1}{C} \sum_{\ell=1}^{n_l} \frac{\left[p_{\ell,n} - p_0 g_\ell\right]^2}{p_0 g_\ell}. \quad (14)$$

The value of the constant C is unknown, but we do not need to know it in order to minimize WSS. We minimize WSS using the same approach as we used to minimize RSS (see above). We find p_0 that minimizes WSS for a given s and μ to be

$$p_{0_{\mathrm{WSS},\min,s_i,\mu_i}} = \sqrt{\frac{\sum_{\ell=1}^{n_l} p_{\ell,n}^2 / g_\ell}{\sum_{\ell=1}^{n_l} g_\ell}}. \quad (15)$$

We show the surface of WSS for s and μ in S22 Fig. Our point estimates for s, μ, and p_0 for EUR and ASN are $s_{\mathrm{EUR}} = 2.4 \times 10^{-4}$, $\mu_{\mathrm{EUR}} = 8.1 \times 10^{-5}$, $p_{0,\mathrm{EUR}} = 5.72 \times 10^{-2}$, and $s_{\mathrm{ASN}} = 1.6 \times 10^{-4}$, $\mu_{\mathrm{ASN}} = 4.1 \times 10^{-5}$, $p_{0,\mathrm{ASN}} = 6.46 \times 10^{-2}$. The estimates of s and μ are close to the estimates obtained using the unweighted sum of squared differences. The estimate of p_0 is higher than the estimate from the unweighted RSS. From our preliminary investigations, we found the estimate of p_0 is sensitive to the choice of weighting, this seems to be due to the high weighting of regions with low $\mathrm{E}[p_{\ell,t}]$ under the weighted RSS.

References

[1] Sankararaman S, Mallick S, Dannemann M, Pruefer K, Kelso J, Paeaebo S, et al. The genomic landscape of Neanderthal ancestry in present-day humans. Nature. 2014 Mar 20;507(7492):354+.

S9 Fig

The scaled RSS surface ($\text{RSS}_{\text{min}} - \text{RSS}$) **for different** s **and** μ **values for EUR and ASN autosomal chromosomes under the single-locus equilibrium model** ($t = \infty$)**.** Each value of the RSS is minimized over p_0, which makes this a profile RSS surface. Regions shaded in orange represent parameter values of higher RSS.

S10 Fig

The scaled RSS surface ($\text{RSS}_{\text{min}} - \text{RSS}$) **for different** s **and** μ **values for EUR and ASN autosomal chromosomes under the single-locus model for** $t = 2000$**.** Each value of the RSS is minimized over p_0, which makes this a profile RSS surface. Regions shaded in orange represent parameter values of higher RSS. Black circles show bootstrap results of 1000 block bootstrap reestimates, with darker circles corresponding to more common bootstrap estimates.

S11 Fig

The scaled RSS surface ($\text{RSS}_{\text{min}} - \text{RSS}$) **for different** s **and** μ **values for EUR and ASN autosomal chromosomes under a multi-locus equilibrium model** ($t = \infty$)**.** Each value of the RSS is minimized over p_0, which makes this a profile RSS surface. Regions shaded in orange represent parameter values of higher RSS.

S12 Fig

The scaled RSS surface ($\text{RSS}_{\text{min}} - \text{RSS}$) **for different** s **and** μ **values for the X chromosome in the ASN population under a single-locus model for** $t = 2000$ **and assuming equal strength of selection in males and females.** Each value of the RSS is minimized over p_0, which makes this a profile RSS surface. Regions shaded in orange represent parameter values of higher RSS. Black circles show bootstrap results of 1000 block bootstrap reestimates, with darker circles corresponding to more common bootstrap estimates.

S13 Fig

The scaled RSS surface ($\text{RSS}_{\text{min}} - \text{RSS}$) **for different** s **and** μ **values for the X chromosome in the EUR population under a single-locus model for** $t = 2000$ **and assuming equal strength of selection in males and females.** Each value of the RSS is minimized over p_0, which makes this a profile RSS surface. Regions shaded in orange represent parameter

values of higher RSS. Black circles show bootstrap results of 1000 block bootstrap reestimates, with darker circles corresponding to more common bootstrap estimates.

S14 Fig

The scaled RSS surface ($\mathrm{RSS}_{\mathrm{min}} - \mathrm{RSS}$) for the X chromosomes as a function of the initial admixture proportion p_0. Results are shown for a model where only the nearest-neighboring exonic site under selection is considered, and for $t = 2000$ generations after Neanderthals split from the EUR (grey) and ASN (pink) populations. Dots and horizontal lines show the value of p_0 that minimizes the RSS and the respective 95% block-bootstrap confidence intervals. Each value of the RSS is evaluated at the values of the selection coefficient (s) and exonic density of selection (μ) given in .

S15 Fig

Fit between our estimates of p_t for bins of different exon density. Genomic regions with low exonic density (low exonic density rank) contain higher average Neanderthal allele frequency in both in Europeans (grey circle) and Asians (pink circle), a pattern recreated in our model. Dashed lines represent the 95% block bootstrap confidence intervals. The length of segments used to create the bins is 2 cM.

S16 Fig

Fit between our estimates of p_t for bins of different exon density. Genomic regions with low exonic density (low exonic density rank) contain higher average Neanderthal allele frequency in both in Europeans (grey circle) and Asians (pink circle), a pattern recreated in our model. Dashed lines represent the 95% block bootstrap confidence intervals. The length of segments used to create the bins is 1.5 cM.

S17 Fig

Fit between our estimates of p_t for bins of different exon density. Genomic regions with low exonic density (low exonic density rank) contain higher average Neanderthal allele frequency in both in Europeans (grey circle) and Asians (pink circle), a pattern recreated in our model. Dashed lines represent the 95% block bootstrap confidence intervals. The length of segments used to create the bins is 0.5 cM. There are 9 bins, rather than 10 bins, in this figure because

there are many 0.5 cM bins with zero exonic sites. Therefore, we collapsed our results together into a smaller number of bins.

S18 Fig

The scaled RSS surface ($\mathrm{RSS}_{\mathrm{min}} - \mathrm{RSS}$) **for different values of** s **and** μ **for EUR and ASN autosomes under a multi-locus equilibrium model** ($t = \infty$)**.** This surface is constructed using windows of 10 cM, but otherwise analogous to . Each value of the RSS is minimized over p_0, which makes this a profile RSS surface. Regions shaded in orange represent parameter values of higher RSS.

S19 Fig

The scaled RSS surfaces ($\mathrm{RSS}_{\mathrm{min}} - \mathrm{RSS}$) **for different values of** s **and** μ **for the X chromosome under a multi-locus equilibrium model** ($t = \infty$)**.** This surface is constructed using windows of 10 cM. Each value of the RSS is minimized over p_0, which makes this a profile RSS surface. Regions shaded in orange represent parameter values of higher RSS.

S20 Fig

The scaled RSS surfaces ($\mathrm{RSS}_{\mathrm{min}} - \mathrm{RSS}$) **for different** s **and** μ **values for EUR and ASN autosomes under a single-locus model** ($t = 2000$). This surface is constructed using the fraction of EUR and ASN alleles at each site with confident Neanderthal calls (a marginal probability of $> 90\%$). Each value of the RSS is minimized over p_0, which makes this a profile RSS surface. Regions shaded in orange represent parameter values of higher RSS.

S21 Fig

Comparison of the variance and the mean frequency of N_1 **obtained from individual-based simulations.** The figure shows 676 circles representing different combinations of r (recombination rate) and s (selection coefficient). Values of r range from 1×10^{-5} (red circle border) to 1×10^{-2} (black border), s ranges from 1×10^{-5} (yellow circle area) to 4×10^{-4} (light blue area). For each parameter combination, the mean and variance of the frequency of N_1 after $t = 2000$ generations was calculated across 1000 independent runs.

S22 Fig

The scaled weighted RSS surface ($\mathrm{RSS}_{\mathrm{min}} - \mathrm{RSS}$) **for different** s **and** μ **values for EUR**

and ASN autosomal chromosomes under the single-locus model for $t = 2000$. Each value of the RSS is minimized over p_0, which makes this a profile RSS surface.

A Table

Minimum RSS parameters for μ, s and p_0 for different models described in S1 Text. Fig 1 in the main text shows an example of $E[p_t]$ for single locus model, $t = 2000$, for part of chromosome 1.

B Table

The 95% bootstrap confidence intervals for μ, s, and p_0 for different models.

C Table

Correlation between the estimated and the observed mean Neanderthal allele frequency for bins created using segments of different sizes.
