# Supplementary material for: The Strength of Selection against Neanderthal Introgression
Source: PLoS Genet. 2016 Nov 8;12(11):e1006340. doi: 10.1371/journal.pgen.1006340 (PMC5100956; doi:10.1371/journal.pgen.1006340)
Supplement: S3 Text — Here, we describe individual-based simulations to investigate whether the difference in population size between Neanderthals and modern humans can account for the selection coefficient (s) and the exonic density of deleterious sites (μ) that we estimated (main text, S2 Text). (PDF) [file pgen.1006340.s003.pdf]

S3 Text

Individual-based Simulations

Ivan Juric, Simon Aeschbacher, Graham Coop

Here, we describe individual-based simulations to investigate whether the difference in population size between Neanderthals and modern humans can account for the selection coefficient (s) and the exonic density of deleterious sites (μ) that we estimated (main text, S2 Text).

Simulation details

Model

To test the plausibility of our estimates of μ and s, we performed individual-based simulations of a likely demographic scenario prior to admixture between Neanderthals and modern humans. Specifically, we assumed that two diploid populations of constant size N_n (Neanderthals) and N_h (modern humans) split from their common ancestral population of size N_a at time 0. These two populations were then simulated forward in time in complete isolation over T_D non-overlapping generations. In each population we simulated a single biallelic locus with alleles A and a such that the fitness of genotypes AA, Aa, and aa is 1, $1-s$, and $(1-s)^2$. Each generation, we draw two parents of each offspring individual at random and with probability proportional to their fitness. Selection in our model is soft, therefore population size is constant each generation. Mutations between a and A and *vice versa* were assumed to occur at rate u per site and generation. We implemented this by introducing a mutation at frequency $1/(2N)$ with probability $2Nu$ per generation, where N is the absolute number of individuals in the respective population (N_n for Neanderthals and N_h for modern humans).

We assumed that allele frequencies in the ancestral population had reached drift–mutation–selection equilibrium prior to the split. Both the Neanderthal and modern human population were therefore initialized by drawing the frequency of allele a from the diffusion approximation to the respective stationary allele-frequency distribution. Specifically, for each run, the probability that the locus is polymorphic in the ancestral population is equal to the probability that a is

found at any frequency between $1/2N_a$ and $1-1/(2N_a)$, which in the diffusion limit is given by

$$P(\text{site is polymorphic}) = 4N_a u \int_{1/2N_a}^{1-1/2N_a} P(x; N_a, s, u) dx, \tag{1}$$

where $P(x; N_a, s, u)$ is the stationary probability density at frequency x. This density is proportional to Eq. (9.3.3) in reference [1],

$$P(x; N_a, s, u) \propto e^{-4N_a s x} x^{4N_a u-1} (1-x)^{4N_a u-1}. \tag{2}$$

If the locus was polymorphic in the ancestral population in a given run, we drew a frequency x of allele a from the stationary distribution in (2); otherwise we set the initial allele frequency to zero. We assumed that the mutation rate u did not change after the split.

In total, we performed R independent runs. For each run we recorded the frequency of the deleterious allele a in the Neanderthal and human population at the end of the simulation. We then computed the difference d between the frequency of a in Neanderthals and humans, $d = \text{freq}_n(a) - \text{freq}_h(a)$. A difference of $d = 1$ implies that the deleterious allele was fixed in Neanderthals and lost in humans, whereas $d = -1$ implies the converse.

Parameter values and settings

We chose the size of the human population to be the same as the one of the ancestral one, and kept them at $N_h = N_a = 10{,}000$. We ran simulations for three different Neanderthal population sizes: $N_n = 500$, 1000, and 2000. These values span a range of Neanderthal effective population sizes proposed and used by others (e.g. [2, 3]). We also used three different mutation rates, $u = 10^{-8}$, 2×10^{-8}, and 3×10^{-8}, to reflect a range of plausible per-nucleotide mutations rates in humans [4]. Note that in our context, the mutation rate should be thought of as a rate of non-synonymous mutation in genic regions to make it an appropriate match for the parameters used in reference [5]. For the runs shown in the paper we kept $u = 1 \times 10^{-8}$, because pilot runs with other values of u did not strongly change our qualitative conclusions. For each parameter set, we simulated 8 million runs. In each simulation run, we drew s from a distribution of scaled selection coefficients proposed by reference [5]. We used their estimated gamma distribution for $2Ns$ (with parameters $\alpha = 0.184$ and $\beta = 8200$), where $N = 25{,}636$ is the effective population size in their model [5].

The timing of admixture between Neanderthals and modern humans relative to the population bottleneck associated with the human exit out of Africa is unclear. To explore the effect of a

bottleneck on the accumulation of genetic load in modern humans, we ran simulations in which the size of the human population prior to admixture was set to $N_h = 14{,}400$. The human population is maintained at this size from the split from Neanderthals until T_b generations before admixture with Neanderthals. The population size then drops to $N_h = 1861$ and is maintained at this number until the time of admixture. We set T_b to 10, 100, and 1000 to reflect our uncertainty about the timing of the bottleneck relative to admixture. The human effective population sizes prior and during the bottleneck were chosen to approximately match those suggested in a previous study [6]. Simulations were stopped at the time of admixture and used to generate the results shown in S26 Fig, S27 Fig, and S28 Fig.

S23 Fig

Simulations showing that the Neanderthal population is predicted to harbor an excess of weakly deleterious fixed alleles compared to humans. (A) A two-dimensional histogram of the difference in allele frequency between the Neanderthal and human population, and the deleterious selection coefficient over all simulated sites. (B) The fraction of sites in the simulations where there is a human- or Neanderthal-specific fixed difference, binned by selection coefficient. Dotted lines indicate the nearly-neutral selection coefficient (i.e. the inverse of the effective population size) for Neanderthal (right) and Human (left) populations. Solid lines show the 95% CI of s for ASN (the larger of the two CI) that we inferred. Note that monomorphic sites are not shown, but are included in the denominator of the fraction of sites. In contrast to Figure 5, $N_n = 500$ and $u = 10^{-8}$.

S24 Fig

Simulations showing that the Neanderthal population is predicted to harbor an excess of weakly deleterious fixed alleles compared to humans. Details are as in S23 Fig, except that $N_2 = 1000$.

S25 Fig

Simulations showing that the Neanderthal population is predicted to harbor an excess of weakly deleterious fixed alleles compared to humans. Details are as in S23 Fig, except that $N_2 = 2000$.

S26 Fig

The Neanderthal population is predicted to harbor an excess of weakly deleterious fixed alleles compared to humans even after a bottleneck. In contrast to S23 Fig, there is a bottleneck in the human population of length $T_b = 10$ generations prior to admixture with Neanderthals. The long-term effective size of the human population prior to the bottleneck was set to $N_h = 14\,400$, and the effective size during the bottleneck to 1861 (see S3 Text for details). Other details are as in S23 Fig.

S27 Fig

The Neanderthal population is predicted to harbor an excess of weakly deleterious fixed alleles compared to humans even after a bottleneck. Details are as in S26 Fig, but the duration of the bottleneck was set to $T_b = 100$ generations.

S28 Fig

The Neanderthal population is predicted to harbor an excess of weakly deleterious fixed alleles compared to humans even after a bottleneck. Details are as in S26 Fig, but the duration of the bottleneck was set to $T_b = 1000$ generations.

References

[1] Crow JF, Kimura M. An introduction to population genetics theory. An introduction to population genetics theory. 1970;.

[2] Pruefer K, Racimo F, Patterson N, Jay F, Sankararaman S, Sawyer S, et al. The complete genome sequence of a Neanderthal from the Altai Mountains. Nature. 2014 Jan 2;505(7481):43+.

[3] Vernot B, Akey JM. Complex history of admixture between modern humans and Neandertals. Am J Hum Genet. 2015 Mar;96(3):448–453.

[4] Segurel L, Wyman MJ, Przeworski M. Determinants of Mutation Rate Variation in the Human Germline. Annual Review of Genomics and Human Genetics. 2014;15:47–70.

[5] Boyko AR, Williamson SH, Indap AR, Degenhardt JD, Hernandez RD, Lohmueller KE, et al. Assessing the evolutionary impact of amino acid mutations in the human genome. PLoS One. 2008 May;4(5).

[6] Gravel S, Henn BM, Gutenkunst RN, Indap AR, Marth GT, Clark AG, et al. Demographic history and rare allele sharing among human populations. Proceedings of the National Academy of Sciences. 2011;108(29):11983–11988.
